# Non-contact incipient fault diagnosis method of fixed-axis gearbox based on CEEMDAN

**DOI:** 10.1098/rsos.170616

**Published:** 2017-08-23

**Authors:** Vanraj, S. S. Dhami, B. S. Pabla

**Affiliations:** Mechanical Engineering Department, National Institute of Technical Teachers Training and Research, Chandigarh 160019, India

**Keywords:** CEEMDAN, condition monitoring, fixed-axis gearbox, sound based fault diagnosis, non-contact diagnosis

## Abstract

Gearbox plays most essential role in the modern machinery for transmitting the required torque along with motion and contributes to wide range of applications. Any failure in gearbox components affects the productivity and efficiency of the system. Most machine breakdowns related to gears are a result of improper operating conditions and loading, hence lead to failure of the whole mechanism. Ensemble Empirical Mode Decomposition (EEMD) comprises advancement and valuable addition in Empirical Mode Decomposition (EMD) and has been widely used in fault detection of rotating machines. However, intrinsic mode functions (IMFs) produced by EEMD often carry the residual noise. Also, the produced IMFs are different in number due to addition of white Gaussian noise, which leads to final averaging problem. To alleviate these drawbacks, Complete Ensemble Empirical Mode Decomposition with Adaptive Noise (CEEMDAN) was previously presented. This paper describes and presents the implementation of CEEMDAN for fault diagnosis of simulated local defects using sound signals in a fixed-axis gearbox. Statistical parameters are extracted from decomposed sound signals for different simulated faults. Results show the effectiveness of CEEMDAN over EEMD in order to obtain more accurate IMFs and fault severity.

## 1. Introduction

With advancement in technology, an increase in system complexity leads to increase in probability of system failure [1]. Over more than three decades, many condition monitoring techniques have been developed which are based on measurement of dynamic responses like acoustics, vibration, eddy current, thermal fields and radiography, acquired by using various sensors which are mounted near the vicinity of the machines [2]. Intelligent fault diagnosis system is aimed to predict the current status of the machines, which facilitate timely preventive steps to increase the reliability with lower maintenance cost.

For gearbox fault diagnosis, vibration is considered as a key component, thus attracting researchers towards acquiring, analysing and quantifying this parameter. A lot of work on condition monitoring and fault diagnosis of fixed-axis gearbox has been reported in the literature; however, only a few have found their way to industrial applications [3]. Many researchers presented the utility of vibration signatures in the anticipation of defects, but only a few emphasized application of sound emission for the detection of faults. However, there is a huge potential to monitor the status of the machines based on sound signals. Many non-contact condition based methodologies such as infrared thermography, laser-based vibration measurement etc. for mechanical systems were presented in the literature which demonstrates the advantages and need of non-contact type measurement in industrial environment [4,5].

Sound based condition monitoring provides some technical advantages over vibration based methods. Firstly, sound sensor can be placed at a distance in either direction or periphery of the monitored system whereas vibration monitoring technique requires surface contact placement in specified direction to get accurate and meaningful information. Secondly, sound monitoring is more sensitive to certain physical processes than surface vibration, hence provides an opportunity to identify faults in early stage [6–8].

Sound signature of the gearbox generally shows a very non-stationary characteristic amid growing faults, thereby challenging to analyse the fault related features. In this context, researchers gained a great attention towards advanced signal processing tools viz. wavelet analysis, Empirical Mode Decomposition (EMD), Ensemble Empirical Mode Decomposition (EEMD), Hilbert huang transform and short time Fourier transform [9]. Complete Ensemble Empirical Mode Decomposition with Adaptive Noise (CEEMDAN) is a very recent signal decomposition technique practically implemented in biomedical signal processing [10,11]. CEEMDAN produces exact reconstruction of a signal by eliminating mode mixing problem which exists in its predecessors such as EMD and EEMD, hence a better spectral separation of mode functions could be achieved. The authors have found that implementation of CEEMDAN in structural health monitoring and machine fault diagnosis is yet to be reported in the literature. Using the strength of CEEMDAN to extract the weak characteristics from noisy signals, a new fault diagnosis method of fixed-axis gearbox based on CEEMDAN is proposed in this paper.

The objectives of this work are:

— To develop a non-contact type fault diagnosis methodology for gearbox by using the advantages of CEEMDAN.
— To develop an intrinsic mode function (IMF) selection methodology for selecting the best sensitive IMF related to fault.

## 2. Theoretical framework

In this section, theoretical background and shortcomings of the EMD and EEMD are explained. A fault diagnosis approach based on CEEMDAN is proposed.

### 2.1. Empirical mode decomposition and ensemble empirical mode decomposition

EMD has proven to be the most powerful signal processing technique with wide applications to the fault diagnosis of rotating machinery [12]. EMD comes under non-stationary, nonlinear signal processing technique which decomposes the signal to get IMFs that are orthogonal in nature and represents the periodic modes clubbed in the signal. The IMFs are not regulated by predetermined kernels; rather these are the basis functions which are determined by the signal itself. A finite number of IMFs can be obtained by decomposing the given signal [13]. EMD has been widely adopted in signal processing, although it possesses mode mixing problem which was overcome by EEMD by introducing the concept of noise-assisted data analysis [14,15]. EEMD defines the IMF as an ensemble average of corresponding IMFs which were decomposed from the original signal with white Gaussian noise addition. Hence EEMD alleviated the problem of mode mixing; however, it leads to creation of some additional problems, i.e. the reconstructed signal includes residual noise along with different realizations of combined signal and noise which result in generation of different number of modes [16–18].

### 2.2. Complete ensemble empirical mode decomposition with adaptive noise

CEEMDAN was proposed to overcome all of the above problems by reconstructing the signal exactly of the original signal along with providing better modes of spectral separation with lower computational cost [16]. It resolved the problem of final averaging. CEEMDAN, being a data-driven approach, does not require a prior basis function, thus facilitating the processing of highly nonlinear and non-stationary signals such as gearbox sound signals.

**Figure 1.**
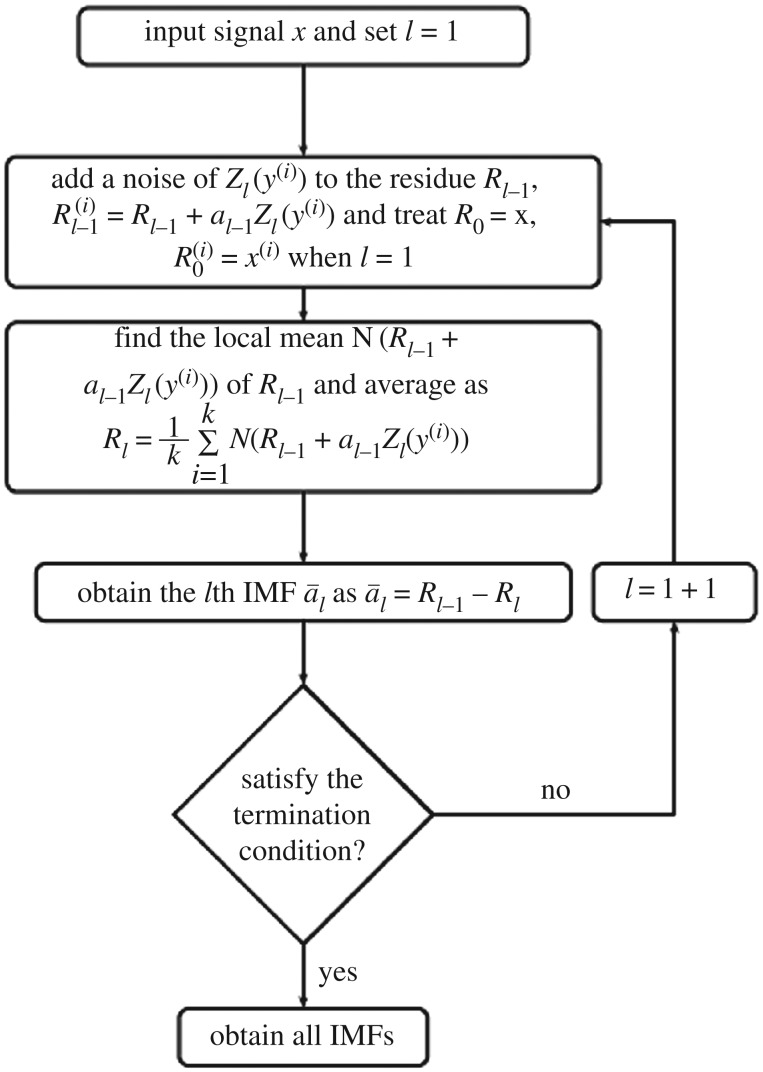
Flow chart of the CEEMDAN algorithm.

For the extraction of *l*th IMF, a particular noise Z*_l_*(*y*^(*i*)^) is added instead of white Gaussian noise. A unique residue is obtained which defines the true IMF equal to difference between the original residue and the average of its local means. Hence the problem of the final averaging because of a different number of IMFs is resolved. The flowchart of the CEEMDAN algorithm is shown in figure 1 and its steps are described as below:

*Step 1.* Addition of *Z*_1_(*y*^(*i*)^) to the original signal *x*, *x*^(*i*)^ = *x* + *α*_0_*Z*_1_(*y*^(i)^), where *y*^(*i*)^ is the *i*th added noise and *α*_1_ represents the selection of the SNR section at each stage computed as *α_l_* = *ϵ*_0_ *std* (*R_l_*)*.*

*Step 2.* Calculation of local means of *x*(*i*) using EMD and find average to calculate first residue

$$R_{1}=\frac{1}{k}\sum_{i=1}^{k}N(x^{i}).$$

Then calculate first IMF ${\tilde{a}}_{1}=x-R_{1}.$

*Step 3.* Calculate the second IMF ${\tilde{a}}_{2}=R_{1}-R_{2}$

where $R_{2}=\frac{1}{k}\sum_{i=1}^{k}N(R_{1}+\alpha_{1}Z_{2}(y^{i}))$

*Step 4.* Similarly, compute *l*th IMF ${\tilde{a}}_{l}=R_{l-1}-R_{l}$

where $R_{l}=\frac{1}{k}\sum_{i=1}^{k}N(R_{l-1}+\alpha_{l-1}Z_{l}(y^{i}))$.

### 2.3. Fault diagnosis based on CEEMDAN

**Figure 2.**
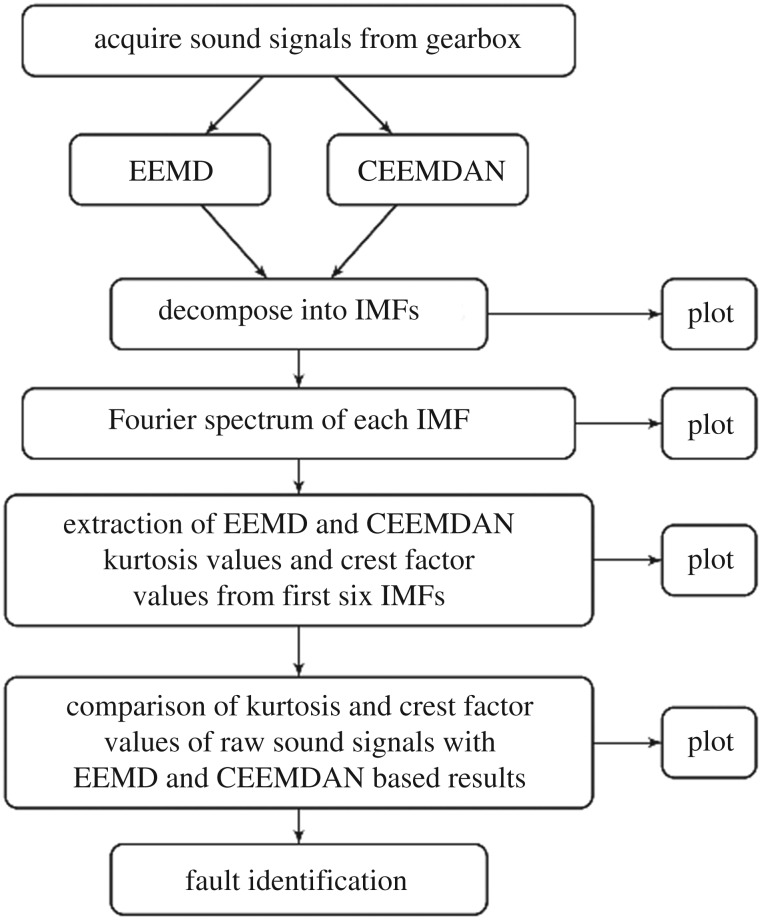
A brief framework of proposed method.

Keeping in mind the superiority of CEEMDAN over EEMD, a frequency domain characteristic extraction method based on CEEMDAN is proposed for fault diagnosis of fixed-axis gearbox. The framework of proposed methodology consists of the following steps, as shown in figure 2. The sound signature is acquired from the gearbox and decomposed into a series of IMFs using CEEMDAN. Fourier spectrum of each IMF is calculated and gear-mesh frequency (GMF) and its harmonics are extracted. Based on the amplitude of GMF, presence of fault and its severity is diagnosed.

## 3. Experimental set-up and data acquisition

**Figure 3.**
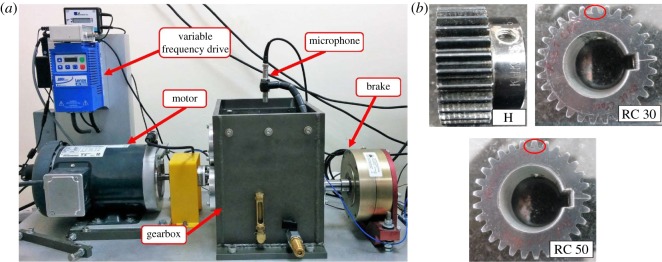
(*a*) Experimental set-up and (*b*) different gear conditions.

Figure 3*a* shows the experimental set-up for condition monitoring of two stage gearbox. The set-up has been designed to simulate real working conditions of a gearbox. The detailed specifications of the test rig are listed in table 1. Table 2 summarizes the specifications of the gearbox.

**Table 1.** Test rig specifications.

parameters	values/description
driving motor	3 HP, 3-phase
variable frequency drive	Lenze
speed measurement	Tachometer
rotational speed	100 r.p.m. to 3600 r.p.m.
external loading type	magnetic loading
loading capacity	0.126 N m to 24.85 N m
speed reduction	single stage with ratio 3.44 : 1

**Table 2.** Gearbox specifications.

specification	values
length	27.5 cm
width	19 cm
height	26.5 cm
no. teeth on pinion	29
no. teeth on gear	100
spur gear pressure angle	20°

For acoustic signal acquisition, microphone with frequency range of 5 Hz to 20 kHz was used. Sound sensor position is important while acquiring acoustic signals. The position of the microphone is fixed at 24 cm vertically from the bottom of the set-up near the meshing pair in accordance to the optimal sensor location experimentation conducted by Vanraj *et al*. [19,20].

Since the time period to overhaul a new gearbox may vary from six months to 1 year depending upon working conditions, naturally generated faults and their detection become difficult. Therefore, the only option left is to study seeded fault trials in gearbox. Most common gearbox faults can be categorized as, (i) root crack (ii) surface spalling and (iii) chipped tooth. The chipped tooth is the rupture of material from the working tip of gear. Root crack is extremely common in numerous industrial practices which triggers other gear faults and is very difficult to detect at its initial stage [2]. Spur gears with simulated root crack damage generated by wire electrical discharge machining in steps based on per cent cutting of teeth root have been considered in the present study. Three types of gear tooth faults are investigated, viz. a healthy gear, gear with 30% and 50% root crack as shown in figure 3*b*. The faults were induced on the pinion mounted on the input shaft. The notations of all running conditions are listed in table 3. For all simulated gear conditions, sound signals are acquired. The sampling rate of the data acquisition system is fixed to 12.8 kHz and 30 000 data points are collected for each case at 2100 r.p.m. with ±30 r.p.m. speed fluctuation under no load. Each test is repeated five times for every fault conditions. Data consists of five files for each kind of fault hence a total of 15 files were used for signal processing. The theoretical characteristic GMF and its harmonics are calculated as GMF (Hz) = (r.p.m. * no. teeth on gear)/60.

**Table 3.** Fault description.

fault	notations
healthy	H
root crack 30%	RC30
root crack 50%	RC50

## 4. Results and discussion

Based on the procedure described in §3, EEMD and CEEMDAN are used to decompose the acquired fault condition sound signals into a number of IMFs ranging from 10 to 12. For illustration, the first six IMFs and their corresponding Fourier spectrum are considered for each fault condition. These six IMFs consist of higher order GMF and its harmonics. Lower frequency IMFs (IMF 7–IMF 12) have been ignored in this case. Higher order statistical parameters i.e. RMS, kurtosis, skewness, crest factor and impulse factor suggested by different studies [3,21–23] are used to evaluate the fault condition of the gearbox. In the present experiment, calculated statistical parameters computed from raw sound signals exhibit irregular trend with respect to fault propagation, thus unable to provide correct information regarding the state of the gearbox. Hence to overcome this drawback, CEEMDAN method is used to mine the features related to fault from sound signals of gearbox.

Kurtosis values of the first six IMFs are calculated for the sound signals obtained from EEMD and CEEMDAN. It is observed that among all IMFs, the values of second IMF of CEEMDAN not only show an increasing trend with increasing fault for all gear conditions but exhibit much higher amplitude than that obtained from raw signals and second IMF of EEMD. It was observed that the raw signal and EEMD fail to diagnose increasing fault severity of the gearbox, whereas kurtosis values of second IMF of CEEMDAN demonstrates useful trends to detect the growing fault conditions even at early stages. Tables 4 and 5 list the values of kurtosis obtained from first six IMFs using EEMD and CEEMDAN respectively. It is observed that for raw signal, a decrease in crest factor value from 4.81 to 4.09 as the severity of the fault increases.

**Table 4.** Kurtosis value of IMFs obtained from EEMD.

	kurtosis values of IMFs					
condition	IMF1	IMF2	IMF3	IMF4	IMF5	IMF6
H	2.42	2.85	2.72	3.47	2.47	3.10
RC30	2.79	3.20	3.22	2.40	2.50	2.04
RC50	2.82	3.31	3.57	3.10	2.52	2.30

**Table 5.** Kurtosis value of IMFs obtained from CEEMDAN.

	kurtosis values of IMFs					
condition	IMF1	IMF2	IMF3	IMF4	IMF5	IMF6
H	2.46	8.70	3.42	3.52	2.62	2.94
RC30	2.77	9.19	5.66	3.22	2.91	2.57
RC50	2.91	11.42	5.10	3.59	4.16	3.29

However, second IMF crest factor values obtained from EEMD show an increasing trend but the values are much less than the raw signal values. CEEMDAN kurtosis values outperform both raw signal and EEMD signals as obtained values are much higher as well as showing clear increasing trend. There is an increase in crest factor value of CEEMDAN second IMF from 5.58 to 6.91. Tables 6 and 7 gives crest factor values obtained from first six IMFs of EEMD and CEEMDAN, respectively.

**Table 6.** Crest factor value of IMFs obtained from EEMD.

	crest factor values of IMFs					
condition	IMF1	IMF2	IMF3	IMF4	IMF5	IMF6
H	2.63	3.11	2.95	3.46	2.34	2.76
RC30	2.81	3.30	3.28	2.71	2.40	1.90
RC50	3.05	3.36	3.55	3.33	2.64	2.34

**Table 7.** Crest factor value of IMFs obtained from CEEMDAN.

	crest factor values of IMFs					
condition	IMF1	IMF2	IMF3	IMF4	IMF5	IMF6
H	2.67	5.58	3.49	3.38	2.57	2.79
RC30	2.82	6.05	4.87	3.21	3.37	2.52
RC50	3.21	6.91	2.24	3.31	4.01	3.01

**Figure 4.**
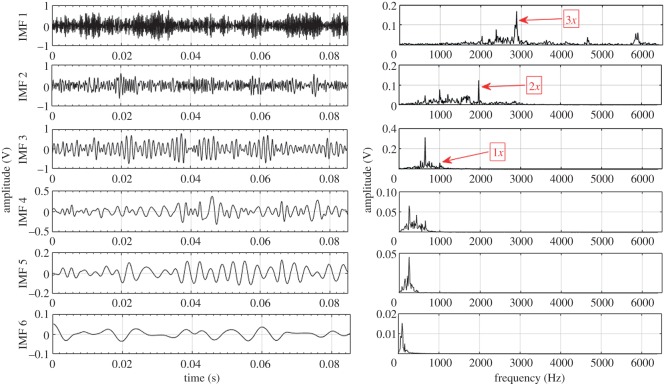
Decomposition results of the sound signal of healthy gear using EEMD.

**Figure 5.**
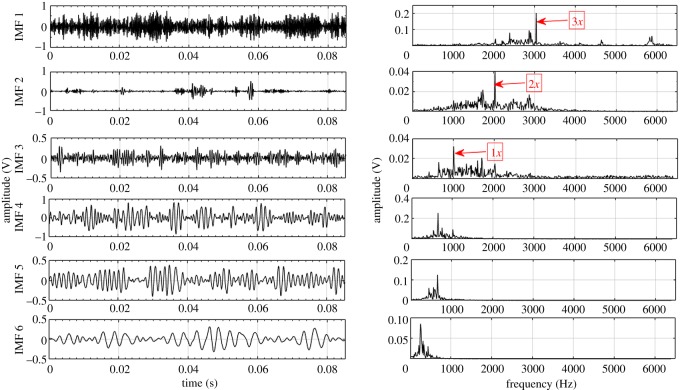
Decomposition results of the sound signal of healthy gear using CEEMDAN.

**Figure 6.**
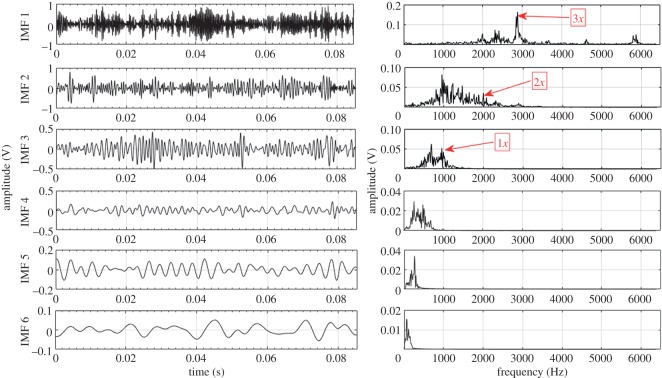
Decomposition results of the sound signal of RC30 using EEMD.

**Figure 7.**
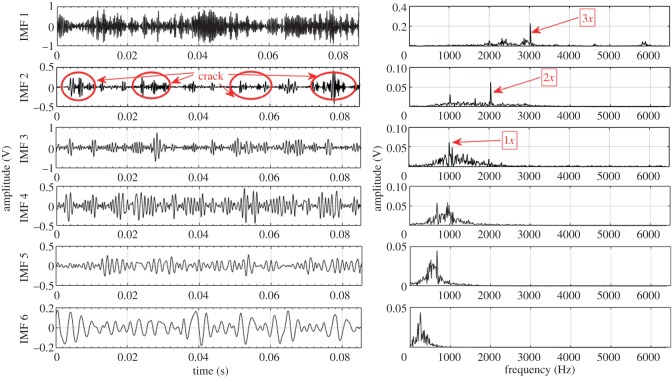
Decomposition results of the sound signal of RC30 using CEEMDAN.

**Figure 8.**
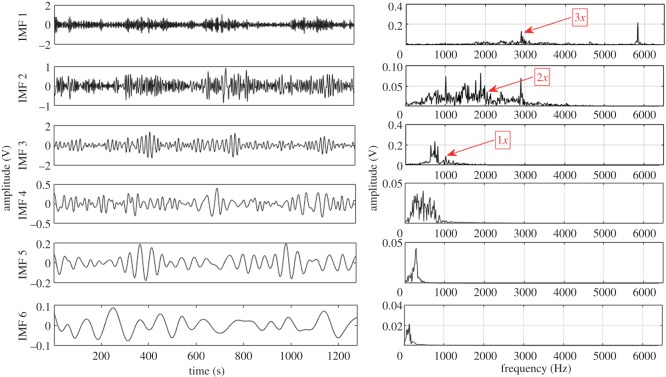
Decomposition results of the sound signal of RC50 using EEMD.

**Figure 9.**
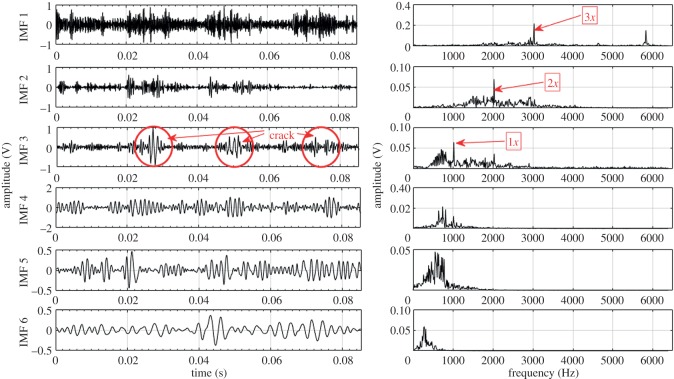
Decomposition results of the sound signal of RC50 using CEEMDAN.

Other higher order statistical parameters are also calculated from EEMD and CEEMDAN based signal processing methods, but the values are too low to be useful in fault detection. Further, to depict the advantage of CEEMDAN over EEMD, capability to separate GMF and its harmonics is also compared. Table 8 shows the measured GMF and its harmonics for each gear condition obtained from IMFs decomposed by EEMD and CEEMDAN. For demonstration purposes, first six IMFs and their corresponding Fourier spectrum for healthy, RC30 and RC50 condition are illustrated in figures 4–9. There is a large variation between theoretical and EEMD detected characteristic frequency because of fluctuation in rotational speed. However, there is a small variation in the case of CEEMDAN. Table 9 shows the amplitude of measured GMF and its harmonics for each gear condition. It is observed that for CEEMDAN, the amplitude shows an increasing trend as the severity of the fault increases. Hence, CEEMDAN can be used to detect the fault condition and its severity in frequency domain. However, EEMD lags in terms of severity detection as there is a random trend.

**Table 8.** Defect frequencies of gearbox extracted from EEMD and CEEMDAN.

		H		RC30		RC50	
GMF (Hz)	theoretical value	EEMD measured	CEEMDAN measured	EEMD measured	CEEMDAN measured	EEMD measured	CEEMDAN measured
1*x*	1015	1012	1014	1010	1014	1012	1014
2*x*	2030	1975	2025	2000	2024	1987	2022
3*x*	3045	2887	3037	2887	3020	2900	3030

**Table 9.** Amplitude of measured GMF and its harmonics extracted from EEMD and CEEMDAN.

		GMF amplitude (V)		
extracted from		1*x*	2*x*	3*x*
EEMD	H	0.0766	0.1224	0.1685
	RC30	0.0821	0.0361	0.1638
	RC50	0.0736	0.0837	0.1344
CEEMDAN	H	0.0322	0.0422	0.2015
	RC30	0.0169	0.0621	0.2276
	RC50	0.0643	0.0692	0.2160

## 5. Conclusion

Experimental study has been carried out on fixed-axis gearbox for fault diagnosis and fault severity on spur gear. Statistical parameters of IMFs extracted from sound signals using CEEMDAN were used for fault detection and severity of faults. To demonstrate the effectiveness of CEEMDAN, a comparison with EEMD is also conducted. An investigation on capability of CEEMDAN for fault severity and detection in frequency domain is also performed. Experimental observations draw following conclusions:

— Initially statistical parameters are extracted for raw sound signals in order to obtain the fault severity. But due to uneven trend these failed to reveal the fault information, thus ignored in this study.
— CEEMDAN based kurtosis and crest factor values provides good diagnosis results due to its capability to decompose signal into different higher to lower frequency modes called IMFs. Hence, it is concluded that the proposed method has the ability to extract the gearbox fault characteristic and diagnose the severity of fault.
— Kurtosis and crest factor values of IMF 2 obtained from CEEMDAN reveal comparatively higher values than raw signal. IMF 2 corresponds to the 2x of gear mesh frequency.
— Kurtosis values of raw and EEMD based IMFs show irregular trends because of speed fluctuation and noise influence.
— Fourier transform of IMFs obtained from CEEMDAN demonstrates that characteristic frequencies can be easily separated as compared to EEMD.
— An increasing trend of amplitude of gear mesh frequency and its harmonics for CEEMDAN with increase in fault severity is obtained. Hence, it is concluded that the frequency domain features obtained from CEEMDAN could be used for fault diagnosis purpose.

## 6. Research trends

Future research may focus on intelligent automatic IMF selection in order to improve the performance and accuracy of the diagnosis method. Also, the performance of CEEMDAN can be verified with data containing large speed fluctuations. Various types of faults (e.g. chipped tooth) should be tested to demonstrate the generalization of CEEMDAN technique in reliable classification of various faults. Authors also suggest to evaluate the robustness of the proposed methodology using field data collected in real world applications. Lastly, artificial intelligent techniques can be employed in combination with CEEMDAN for accurate classification results.

## Data accessibility

Dryad Digital Repository: http://dx.doi.org/10.5061/dryad.s4g56 [24].
